# Langer-Giedion Syndrome: a Rare Case Report

**Published:** 2016-09

**Authors:** Farhin Ali Katge, Bhavesh Dahyabhai Rusawat, Pooja Ravindra Shivasharan, Devendra Pandurang Patil

**Affiliations:** 1 Dept. of Pedodontics & Preventive Dentistry, Terna Dental College, Navi Mumbai, Maharashtra, India.; 2 Postgraduate Student, Dept. of Pedodontics & Preventive Dentistry, Terna Dental College, Navi Mumbai, India.

**Keywords:** Trichorhinophalangeal Syndrome type 2, Exostosis, Hypodontia, Langer-Giedion Syndrome

## Abstract

Langer-Giedion syndrome is a very uncommon autosomal dominant genetic disorder caused by the deletion of chromosomal material. It is characterized by multiple bony exostosis, short stature, mental retardation, and typical facial features. The characteristic appearance of individuals includes sparse scalp hair, rounded nose, prominent philtral area and thin upper lip. Some cases with this condition have loose skin in childhood which typically resolves with age. Oral and dental manifestations include micrognathia, retrognathia, hypodontia, and malocclusion based on cephalometric analysis. This report presents a case of Langer-Giedion syndrome in a 10-year-old child.

## Introduction

Trichorhinophalangeal syndrome (TRPS) type 2, also known as Langer-Giedion syndrome, is a rare gene deletion syndrome with distinct facial features and bone abnormalities.[1] Most of the cases are sporadic but father-to-son and mother-to-daughter transmission has been documented.[2-5]The skeletal structure shows multiple exostoses in the long and short tubular bones of the limbs. This is a differentiating feature between TRPS 1 and 2.[6-8] The craniofacial features include bulbous nose, prominent philtral area, thin vermillion of upper lip, sparse scalp hair, prominent forehead, mild microcephaly and broad eyebrows.[5, 9] Oral manifestations include micrognathia, retrognathia, supernumerary teeth, hypodontia, and malocclusion. The infants experience feeding problems due to uncoordinated swallowing causing choking.[5] Other manifestations include loose skin, recurrent respiratory tract and middle ear infections during childhood.[2, 5, 8] This report describes the clinical manifestations and dental management of a patient with Langer-Giedion syndrome.

## Case Report

A 10-year-old female patient with a known medical history of Langer-Giedion syndrome was reported to the Department of Pedodontics and Preventive Dentistry, Terna Dental College, Navi Mumbai, India with the chief complaint of pain in upper left posterior region of the mouth for 6 months. The pain was continuous and throbbing in nature. It was aggravated on drinking cold water, eating sweets and was relieved on taking analgesics prescribed by the child’s physician. The patient had never visited a dentist before. The diagnosis of the Langer-Giedion syndrome had previously been established on the basis of the facial, hair, nasal and digital features at the age of seven. Moreover, the cytogenetic evaluation of G-banded metaphases revealed normal female karyotype. A detailed family history given by mother was not medically relevant with respect to the syndrome or related symptoms. It did not reveal any consanguinity in the family. Natal history of the patient revealed that the child had jaundice at birth and had loose skin. Birth weight of the patient was 1.5 kilograms (kg). She was not breastfed as there was difficulty in suckling, and was, thus, fed by breast milk with the help of a syringe for 6 months. All the vaccines were given as per the schedule.

**Figure 1:**
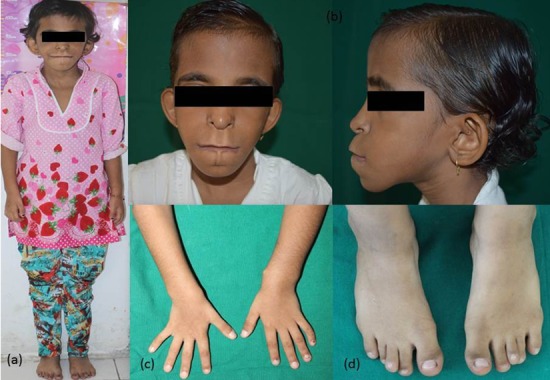
General features: a: Short stature. b: Sparse scalp hair, outstanding pinnae, prominent philtral area, thin vermillion of upper lip. c: Bony exostosis. d: Overlapping toes.

The patient presented with characteristics of the syndrome. She had an abnormal short stature with a weight of 11 kg and height of 106 centimetres. On examination, she presented with anomalies such as multiple bony exostoses on the body, brachydactyly, and overlapping of the toes. The craniofacial features seen were sparse scalp hair, broad forehead, pear shaped nose, outstanding pinnae, prominent philtral area, thin vermillion of upper lip, and prominent mandibular symphyseal region resulting in an ape-like appearance (Figure 1).

**Figure 2:**
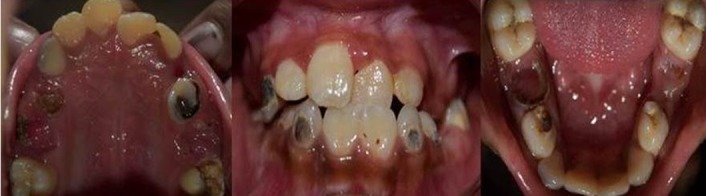
Intraoral preoperative photographs

**Figure 3:**
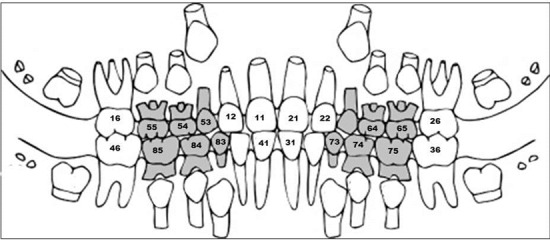
Chart showing tooth numbering

A firm swelling was seen on the gingiva in the 64, 65 region. Dental age of the patient was eight to nine years. Root pieces of 54, 55, 64, 65, 75 and 85 were seen. Pit and fissure caries were present with 16, 36, 46 and 84. Smooth surface caries was present with 53, 73 and 83. Anterior crossbite was present with 21 (figures 2 and 3). Mild supragingival calculus and bleeding on probing was reported on examination.

**Figure 4:**
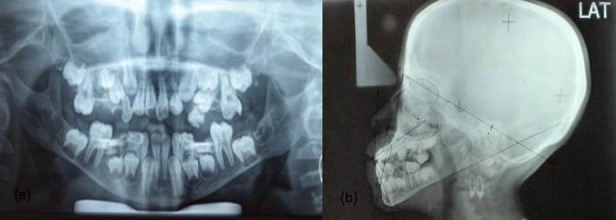
Extraoral radiographs: a: Orthopantomogram, b: Cephalometric tracing (Steiner’s analysis)

The patient was advised for orthopantomogram (OPG), lateral cephalogram, and intraoral periapical (IOPA) radiograph. The OPG revealed multiple decayed teeth and congenitally missing 32 and 42 (Figure 4a). Steiner’s cephalometric analysis revealed retrognathic maxilla and mandible (Figure 4b). Soft tissue analysis suggested convex profile. Routine blood investigations were performed which included bleeding time, clotting time, prothrombin time (PT) and partial thromboplastin time (PTT) to rule out any bleeding or clotting disorder. The values were within normal limits.

**Figure 5:**
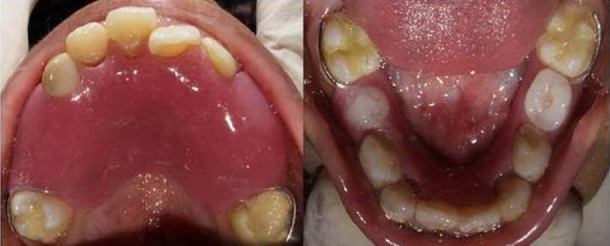
Post-operative photographs

The patient was referred to the Paediatrician for overall examination and assessment of the condition in particular. Written consent was obtained from the Paediatrician for performing the dental extractions. Preparatory treatment included oral prophylaxis followed by root canal treatment was done with 26. Restoration was done for 73, 74, 83 and 84 by using Glass Ionomer cement type IX (GC Corporation Tokyo; Japan). Composite restorations (SDI Limited; Australia) were done for 53, 16, 36 and 46. Extraction of root pieces in relation to 54, 55, 64, 65, 75 and 85 were carried out. Removable non-functional space maintainer was delivered for maxillary arch; whereas, functional space maintainer was delivered for mandibular arch (Figure 5). In the maintenance phase, the patient was instructed for proper brushing technique and fluoridated toothpaste was also advised as a preventive measure.

## Discussion

The case presented above was a sporadic case of Langer-Giedion syndrome with no history of the syndrome in the family. Diagnosis was based on clinical findings and was confirmed by cytogenetic testing reports which showed deletion of TPRS1 and exostosin glycosyltransferase 1 (EXT 1) gene which is localized to 8q24.11 - 8q24.13 region

Langer-Giedion syndrome can affect the growing child psychologically, socially and esthetically. In this presented case, the condition was considered to be a social stigma by the relatives. The mild intellectual disability compounded a lack of co-operation and made the assessment difficult. However, intra-oral examination revealed evidence of inadequate oral hygiene and dental caries. Hence, parental counselling is a very essential component in the management of this syndrome. Early dental evaluation and regular follow-up after treatment must be carried out. The life expectancy of these patients is long, although mental retardation may possess a major problem in some of the cases. A few cases have also been reported with submucous cleft palate.[10]

Differential diagnoses include Ehlers-Danlos syndrome because of excessive redundancy and looseness of skin especially at birth and during early infancy and osteochondromatosis syndrome or Ollier’s disease, if the striking facial features of Langer-Giedion syndrome are absent.[1, 5]

The most outstanding clinical presentation of a case of Langer-Giedion syndrome is the unique facial features and the short stature. No definite treatment regimen has been reported for esthetic correction.

Further research is required to establish a protocol for esthetic correction in these patients. At present, a long-term follow-up throughout the life is essential in dental management of this syndrome.

Dental care is becoming recognised as integral to the overall management of patients with genetic disorders. On this basis it can be expected that heritable conditions such as Langer-Giedion syndrome will increasingly be referred to academic dental facilities for specialised appraisal and care. From this perspective, we have documented our own experience. 

## References

[1] Lüdecke HJ, Wagner MJ, Nardmann J, La Pillo B, Parrish JE, Willems PJ (1995). Molecular dissection of a contiguous gene syndrome: localization of the genes involved in the Langer-Giedion syndrome. Hum Mol Genet.

[2] Hall BD, Langer LO, Giedion A, Smith DW, Cohen MM Jr, Beals RK (1974). Langer-Giedion syndrome. Birth Defects Orig Artic Ser.

[3] Keret D, Bar-Maor JA, Reis DN (1984). The Ale-Calo syndrome in monozygotic twins associated with bilateral cryptorchidism--case report. Z Kinderchir.

[4] Kozlowski K, Harrington G, Barylak A, Bartoszewica B (1977). Multiple exostoses-mental retardation syndrome (Ale-Calo or M.E.M.R. syndrome). Clin Pediatr (Phila).

[5] Langer LO Jr, Krassikoff N, Laxova R, Scheer-Williams M, Lutter LD, Gorlin RJ (1984). The tricho-rhino-phalangeal syndrome with exostoses (or Langer-Giedion syndrome): four additional patients without mental retardation and review of the literature. Am J Med Genet.

[6] Buhler EM (1982). Langer-Giedion syndrome and 8q- deletion. Am J Med Genet.

[7] Fukushima Y, Kuroki Y, Izawa T (1983). Two cases of the Langer-Giedion syndrome with the same interstitial de-letion of the long arm of chromosome 8: 46, XY or XX, del (8) (q23.3q24.13). Hum Genet.

[8] Wilson WG, Wyandt HE, Shah H (1983). Interstitial deletion of 8q. Occurrence in a patient with multiple exostoses and unusual facies. Am J Dis Child.

[9] Gericke GS, Fialkov J (1980). The Langer-Giedion phenotype associated with a unique skeletal finding in a mentally retarded adolescent male. A case report. S Afr Med J.

[10] Morioka D, Suse T, Shimizu Y, Ohkubo F, Hosaka Y (1999). Langer-Giedion syndrome associated with submucous cleft palate. Plast Reconstr Surg.

